# Grupos de investigación: juntos llegamos lejos

**DOI:** 10.21142/2523-2754-0903-2021-066

**Published:** 2021-10-06

**Authors:** Angela Quispe-Salcedo

**Affiliations:** 1 Division of Anatomy and Cell Biology of the Hard Tissue. Niigata University Graduate School of Medical and Dental Sciences, Niigata, Japan. aquispesa@cientifica.edu.pe, aquispesa@dent.niigata-u.ac.jp Division of Anatomy and Cell Biology of the Hard Tissue Niigata University Graduate School of Medical and Dental Sciences Niigata Japan aquispesa@cientifica.edu.pe aquispesa@dent.niigata-u.ac.jp

En los últimos años, ha sido palpable el esfuerzo para fomentar el desarrollo de la investigación científica en el país, especialmente dentro de las diferentes instituciones de educación superior. Desde el inicio de la reforma universitaria, los lineamientos delimitados por el Consejo Nacional de Ciencia, Tecnología e Innovación Tecnológica (Concytec) han contribuido al avance vertiginoso de la producción científica de las universidades y otros centros de formación, lo que ha cambiado el paradigma de “universidad que enseña” hacia el de “universidad que investiga”. 

Si bien es cierto que lo anterior ha sido posible gracias al incremento del presupuesto para la investigación, canalizándolo a través de fondos concursables para el desarrollo de proyectos en áreas específicas; otras acciones, como la conformación de grupos de investigación en cada una de las instituciones de educación superior, han sido determinantes para fortalecimiento de la investigación científica en nuestro país. Según Concytec, un grupo de investigación se define como “unidades básicas de organización de las actividades de investigación científica y desarrollo tecnológico (I+D) de la universidad, del instituto de investigación o de otras instituciones públicas o privadas pertenecientes al Sistema Nacional de Ciencia y Tecnología (SINACYT) dedicadas a las actividades de investigación” ^1^. Asimismo, señalan que los grupos de investigación “están definidos por un conjunto de personas que conforman un equipo para realizar investigación en una temática determinada, que incluye una o más disciplinas relacionadas” ^1^^,^^2^, es decir, pueden estar enfocados en una temática en particular o, por el contrario, pueden tomar el carácter de multidisciplinarios. 

Para ser parte de un grupo, no es necesario conocer a profundidad las líneas de investigación que lo caracterizan. Lo importante es contar con la motivación y el compromiso suficientes para aportar a los proyectos establecidos, actuando siempre con honestidad y responsabilidad. De aquí la importancia de fomentar el desarrollo sostenido de los grupos de investigación, ya que con la participación de estudiantes y docentes se convierte en un “centro de formación” donde todos pueden aportar y aprender en el proceso. El líder del grupo debe contar con experiencia en el tema para ser la guía que los demás miembros necesitan y apoyarse en los docentes colaboradores para conseguir el avance de los proyectos de investigación, a través de la elaboración de un plan de acción, con metas y objetivos definidos y realizables, sin descuidar el rol de los estudiantes de pre o posgrado, y los tesistas que también son beneficiados con proyectos pequeños en paralelo ^3^.

Cada institución es responsable por el fortalecimiento y el futuro de los grupos de investigación. Aunque, idealmente, todos los grupos deberían ser sostenibles en el tiempo y favorecer la producción científica de la institución, lamentablemente, no todos llegarán a esa meta. Diversas razones, como la falta de renovación de sus integrantes, el poco compromiso de sus miembros y, sobre todo, la escasez de financiamiento para el desarrollo de los proyectos de investigación, hacen peligrar el futuro de los grupos más débiles, conformados usualmente por investigadores noveles, frente a los grupos de excelencia, con historial de financiamiento y amplia producción científica. A fin de aliviar esta situación, las instituciones deberían promover el establecimiento de redes colaborativas entre los grupos de investigación, para incentivar el acercamiento a los grupos de excelencia, inclusive con aquellos pertenecientes a otras instituciones locales. Los concursos de subvenciones económicas podrían establecerse mediante categorías, con apoyos económicos pequeños, accesibles para los grupos de investigación más jóvenes, a fin de que puedan fortalecerse y continuar su crecimiento académico ^3^^,^^4^.

Nuestra universidad, a través de la Dirección General de Investigación, Desarrollo e Innovación (DGIDI), viene promoviendo activamente la inscripción de nuevos grupos de investigación. Hace poco, se ha oficializado la conformación del primer grupo propio de la Carrera de Estomatología y otros más vienen en camino, para sumarse a los 16 grupos ya establecidos y categorizados por la Universidad Científica del Sur. 

Un grupo de investigación no es solo un conjunto de personas que se reúnen para sacar adelante un proyecto. Mediante el trabajo en equipo se transmiten no solo el conocimiento académico, sino también valores y ética profesional, con lo que se convierte en una manera auténtica de promover el desarrollo de cada miembro, a través de la creación de conocimiento, la labor docente y la formativa.
